# Foot-and-Mouth Disease Virus Viroporin 2B Antagonizes RIG-I-Mediated Antiviral Effects by Inhibition of Its Protein Expression

**DOI:** 10.1128/JVI.01310-16

**Published:** 2016-11-28

**Authors:** Zixiang Zhu, Guoqing Wang, Fan Yang, Weijun Cao, Ruoqing Mao, Xiaoli Du, Xiangle Zhang, Chuntian Li, Dan Li, Keshan Zhang, Hongbing Shu, Xiangtao Liu, Haixue Zheng

**Affiliations:** a State Key Laboratory of Veterinary Etiological Biology, National Foot and Mouth Diseases Reference Laboratory, Key Laboratory of Animal Virology of Ministry of Agriculture, Lanzhou Veterinary Research Institute, Chinese Academy of Agricultural Sciences, Lanzhou, China; b Collaborative Innovation Center for Viral Immunology, Medical Research Institute, Wuhan University, Wuhan, China; University of Iowa

## Abstract

The role of retinoic acid-inducible gene I (RIG-I) in foot-and-mouth disease virus (FMDV)-infected cells remains unknown. Here, we showed that RIG-I inhibits FMDV replication in host cells. FMDV infection increased the transcription of RIG-I, while it decreased RIG-I protein expression. A detailed analysis revealed that FMDV leader proteinase (L^pro^), as well as 3C proteinase (3C^pro^) and 2B protein, decreased RIG-I protein expression. L^pro^ and 3C^pro^ are viral proteinases that can cleave various host proteins and are responsible for several of the viral polyprotein cleavages. However, for the first time, we observed 2B-induced reduction of host protein. Further studies showed that 2B-mediated reduction of RIG-I is specific to FMDV, but not other picornaviruses, including encephalomyocarditis virus, enterovirus 71, and coxsackievirus A16. Moreover, we found the decreased protein level of RIG-I is independent of the cleavage of eukaryotic translation initiation factor 4 gamma, the induction of cellular apoptosis, or the association of proteasome, lysosome, and caspase pathways. A direct interaction was observed between RIG-I and 2B. The carboxyl-terminal amino acids 105 to 114 and amino acids 135 to 144 of 2B were essential for the reduction of RIG-I, while residues 105 to 114 were required for the interaction. These data suggest the antiviral role of RIG-I against FMDV and a novel antagonistic mechanism of FMDV that is mediated by 2B protein.

**IMPORTANCE** This study demonstrated that RIG-I could suppress FMDV replication during virus infection. FMDV infection increased the transcriptional expression of RIG-I, while it decreased RIG-I protein expression. FMDV 2B protein interacted with RIG-I and induced reduction of RIG-I. 2B-induced reduction of RIG-I was independent of the induction of the cleavage of eukaryotic translation initiation factor 4 gamma or cellular apoptosis. In addition, proteasome, lysosome, and caspase pathways were not involved in this process. This study provides new insight into the immune evasion mediated by FMDV and identifies 2B as an antagonistic factor for FMDV to evade the antiviral response.

## INTRODUCTION

Foot-and-mouth disease virus (FMDV) is a single-stranded positive-sense RNA virus that causes foot-and-mouth disease (FMD) in cattle, pigs, and various cloven-hoofed animals (1). FMDV genome consists of a 5′ untranslated region (UTR), an integral open reading frame (ORF), and a 3′ UTR with a poly(A) tail. The ORF encodes a polyprotein, which is subsequently proteolysed into at least 13 proteins, such as VP1, VP2, VP3, VP4, leader proteinase (L^pro^), 2A, 2B, 3A, 3B1, 3B2, 3B3, 3C^pro^, and 3D^pol^ (2, 3). FMDV 2B protein is a nonstructural protein that is involved in the rearrangement of host cell membranes and disruption of the cellular secretory pathway (4, 5). FMDV 2B is an ∼17-kDa protein comprising 154 amino acids. Two hydrophobic domains are identified in the N terminus of 2B, which is thought to tether 2BC to the endoplasmic reticulum (ER) (5). A bioinformatics analysis implies that the carboxyl-terminal region of 2B is involved in membrane interaction, which is important for virus replication (6). The 2B protein of other picornaviruses is reported to be involved in virus-induced cytopathic effects, blocking cellular protein secretion and impairing apoptotic responses during virus infection (7–9), whereas the multiple accessory functions of FMDV 2B during viral infection remain unclear.

Retinoic acid-inducible gene I (RIG-I) is a pattern recognition receptor (PRR) that is essential for sensing invading pathogens and initiating the innate immune response (10). RIG-I is activated by infection with various RNA viruses. Activation of RIG-I is responsible for the induction of type I interferon (IFN) and the expression of many cytokines and chemokines. The caspase activation and recruitment domains of RIG-I interact with virus-induced signaling adapter (VISA) and then recruits TANK-binding kinase 1 (TBK1) and TNF receptor-associated factor 6, which finally induce the expression of type I IFNs and inflammatory cytokines through activation of IFN-regulatory factor 3 (IRF3), IRF7, and nuclear factor-κB (NF-κB) transcription factors (11). The secreted type I IFNs subsequently transmit signals to cognate IFN receptors and induce expression of various IFN-inducible genes to initiate an antiviral response (12). In addition to the canonical PRR function, RIG-I can also directly function as an antiviral effector in the absence of IFN signaling (13, 14).

RIG-I recognizes a variety of RNAs from influenza A virus (IAV), paramyxoviruses, Sendai virus (SeV), vesicular stomatitis virus, and hepatitis C virus (15, 16), whereas the sensing of picornavirus RNA is primarily mediated by melanoma differentiation-associated protein 5 (MDA5) (17, 18). Whether RIG-I functions as a viral sensor during FMDV infection remains unclear; however, it is believed that RIG-I also plays a role during picornavirus infection (19). RIG-I is cleaved during poliovirus, rhinovirus, echovirus, and encephalomyocarditis virus (EMCV) infections, and viral proteinase 3C^pro^ induces this cleavage (19). The cleavage of RIG-I possibly contributes to the attenuated antiviral responses.

Despite the cleavage of RIG-I in several picornaviruses, RIG-I is speculated to have different roles in different picornavirus infections (19-22), and little is known about the state and function of RIG-I in FMDV-infected cells. The present study determined the antiviral activity of RIG-I and the state of RIG-I during FMDV infection. FMDV 2B protein showed a novel property of inducing reduction of RIG-I. Therefore, we demonstrated the antiviral role of RIG-I against FMDV and explored a novel antagonistic mechanism of FMDV.

## MATERIALS AND METHODS

### Cells, viruses, and infection.

Porcine kidney PK-15 cells, baby hamster kidney-21 (BHK-21), and human embryonic kidney 293T (HEK293T) cells were cultured in Dulbecco modified Eagle medium (Gibco) supplemented with 10% heat-inactivated fetal bovine serum (FBS; Gibco) and maintained at 37°C (5% CO_2_). FMDV type O strain O/BY/CHA/2010 and type Asia 1 isolate Asia1/HN/2006 were used for viral challenge. The viruses were propagated in BHK-21 cells. Viral infection experiments were carried out as described previously (23). The 50% tissue culture infective dose (TCID_50_) values were determined using the Reed-Muench method. The SeV strain used in this study and its infection method have been described previously (24).

### Plasmids and antibodies.

The cDNA of porcine RIG-I were amplified from PK-15 cells and cloned into pcDNA3.1/myc-His(−)A vector (Invitrogen) to yield the Myc-tagged expression construct (Myc-RIG-I). Each of FMDV full-length viral cDNA was inserted into p3xFLAG-CMV-7.1 vector (Sigma-Aldrich) to construct plasmids expressing Flag-tagged viral proteins. A series of Flag-tagged truncated 2B constructs were generated by site-directed mutagenesis PCR. All constructed plasmids were analyzed and verified by DNA sequencing. The IFN-β promoter luciferase reporter plasmids and various hemagglutinin (HA)-tagged plasmids used in this study were kindly provided by Hongbing Shu (Wuhan University, China) (25). The commercial antibodies used in this study include: anti-Myc monoclonal antibody (Santa Cruz Biotechnology), anti-Flag monoclonal antibody (Santa Cruz Biotechnology), anti-Flag polyclonal antibody (Sigma-Aldrich), anti-RIG-I polyclonal antibody (Abcam), anti-HA tag antibody (BioLegend), anti-eukaryotic translation initiation factor 4 gamma (eIF4G) polyclonal antibody (Abcam), and anti-β-actin monoclonal antibody (Santa Cruz Biotechnology). Anti-VP1 polyclonal antibody was prepared by our laboratory (unpublished data).

### Establishment of an RIG-I knockout PK-15 cell line using the CRISPR/Cas9 system.

The pX330 plasmid expressing Cas9 (Addgene, plasmid 42230) was digested with BbsI (NEB) and ligated to annealed single guide RNA (sgRNA) oligonucleotides targeting porcine RIG-I. The sgRNA sequence used in this study was designed using the online CRISPR design tool (http://crispr.mit.edu/), and the sequence was 5′-GCGGAATCTGCACGCTTTCG-3′. PK-15 cells were seeded into 12-well plates at a density of 3 × 10^5^ cells, and the monolayer cells were transfected with the constructed plasmid (2 μg in each well) for 72 h prior to genomic DNA extraction. For PCR analysis of RIG-I genomic DNA, total genomic DNA was extracted using DNeasy blood and tissue kit (Qiagen) according to the manufacturer's protocol. The DNA pellet was dried and resuspended in double-distilled water. The genomic region surrounding the CRISPR target site was amplified by PCR using the check primers (forward, 5′-AAGTGGTTACACCGCATACA-3′; reverse, 5′-CACCTCAAACTCCGACAATC-3′), and the products were purified and reannealed as described previously (26). T7 endonuclease I (NEB) was used to confirm the genome editing of RIG-I. Digested DNA was separated and analyzed on a 1.5% agarose gel. After confirmation of the activity of the designed sgRNA, the transfected PK-15 cells were plated by limiting dilution, and the RIG-I knockout PK-15 cell line was established by the limiting dilution method in 96-well plates (0.5 cell each well). The genomic DNA of the cells cultured from a single-cell clone was amplified using the check primers. The PCR products were purified and ligated into pMD-18T vectors; six plasmids for each sample were sequenced to ensure the frameshifting mutation of both alleles of the established cell line. A Western blot analysis was performed to confirm that RIG-I was not expressed in the RIG-I knockout cell line; wild-type (WT) PK-15 cell was used as a control.

### Coimmunoprecipitation and Western blot analysis.

HEK293T cells were cultured in 10-cm dishes, and the monolayer cells were cotransfected with various plasmids. The collected cells were then lysed and immunoprecipitated as described previously (27). For Western blotting, target proteins were resolved by SDS-PAGE and transferred onto an Immobilon-P membrane (Millipore). The membrane was blocked and incubated with appropriate primary antibodies and secondary antibodies as described previously (28). The antibody-antigen complexes were visualized using enhanced chemiluminescence detection reagents (Thermo). For the coimmunoprecipitation assay to detect the interaction between RIG-I and 2B, the highly sensitive SuperSignal West Femto maximum sensitivity substrate kit (Thermo), which can detect extremely low amounts of proteins, was used to visualize the antibody-antigen complexes.

### Indirect immunofluorescence microscopy.

HEK293T cells (3 × 10^5^) were grown on Nunc glass-bottom dishes. At 24 h posttransfection (hpt), the cells were covered by acetone-methanol mixture (1:1) for fixation at −20°C (10 min). The specimens were blocked in the blocking buffer (5% normal goat serum in PBS) for 1 h at 37°C. The cells were then incubated with anti-Myc and anti-Flag primary antibodies overnight at 4°C. The specimens were next incubated with fluorochrome-conjugated secondary antibodies in the dark for 1 h at room temperature. After incubation with secondary antibodies, the cells were covered with DAPI (4′,6′-diamidino-2-phenylindole) for 10 min to stain the nuclei. The stained cells and fluorescence were visualized using a Nikon eclipse 80i fluorescence microscope with appropriate settings. The microscopy images were processed using NIS Elements F 2.30 software.

### RNA extraction and qPCR.

Total RNAs were extracted using TRIzol Reagent (Invitrogen). The cDNA was synthesized from the extracted RNA samples, using the M-MLV reverse transcriptase (Promega) and random hexamer primers (TaKaRa). The generated cDNA was used as the template to detect expression of FMDV RNA and host cellular mRNA. The Mx3005P QPCR system (Agilent Technologies) and SYBR Premix ExTaq reagents (TaKaRa) were used in the quantitative PCR (qPCR) experiment to quantify the abundance of various mRNAs. The glyceraldehyde-3-phosphate dehydrogenase (GAPDH) gene was determined as an internal control. Relative expression of mRNA was calculated using the comparative cycle threshold (2^−ΔΔ*CT*^) method (29). All the experiments were repeated three times with similar results. The data represent results from one of the triplicate experiments (*, *P* < 0.05 [considered significant]; **, *P* < 0.01 [considered highly significant]).

### Flow cytometric analysis of apoptosis by annexin V-FITC/PI staining.

Annexin V-propidium iodide (AnnV-PI) staining assay using flow cytometry was performed to evaluate 2B-induced apoptosis in PK-15 cells. A total of 5 × 10^5^ cells were grown in each well of six-well plates. The monolayer cells were transfected with 2 μg of empty vector or 2 μg of Flag-2B-expressing plasmid using Lipofectamine 2000 or infected with FMDV (MOI of 0.05). The supernatants were collected, and the adherent cells were detached with trypsin, which did not contain EDTA and Phenol Red, at 24 hpt. The detached cells and supernatants were mixed, and the whole cells were collected. The collected cells were washed with PBS and resuspended in diluted binding buffer at a density of 10^6^ cells/ml. Flow cytometry was used to assess the loss of membrane asymmetry and integrity by AnnV-PI staining, as described previously (30). Surface exposure of phosphatidylserine on apoptotic cells was detected by AnnV-FITC staining. PI binds with DNA in late apoptotic cells and necrotic cells.

### Proteasome, lysosome, and caspase inhibitor assay.

The proteasome inhibitor MG132 was purchased from Merck & Co; the lysosome inhibitor chloroquine diphosphate (CQ) and the caspase inhibitor benzyloxycarbony (Cbz)-l-Val-Ala-Asp (OMe)-fluoromethylketone (Z-VAD-FMK) were purchased from Sigma. PK-15 cells were cultured in six-well plates to a confluence of 60 to 70% and then incubated with FMDV or serum-free medium for 1 h. After the incubation, the cells were maintained in fresh medium supplemented with 1% FBS in the presence or absence of MG132 (2 or 20 μM), CQ (50 or 100 μM), or Z-VAD-FMK (10 or 50 μM) for 12 h and then collected for Western blotting. As for the HEK293T cells, the cells were cotransfected with Myc-RIG-I-expressing plasmid and Flag-2B-expressing plasmid or empty vector using Lipofectamine 3000 and maintained in the presence or absence of MG132 (2 or 20 μM), CQ (50 or 100 μM), or Z-VAD-FMK (10 or 50 μM) for 36 h. The collected cells were then subjected to Western blotting.

### MTS assay.

An MTS [3-(4,5-dimethylthiazol-2-yl)-5-(3-carboxymethoxyphenyl)-2-(4-sulfophenyl)-2H-tetrazolium] assay was performed to evaluate the cytotoxicity of MG132, CQ and Z-VAD-FMK on PK-15 cells. PK-15 cells (10^3^ cells in each well) were seeded in 96-well plate and cultured in the presence or absence of various inhibitors for 36 h. Cell viability was determined using a Cell Titer 96 AQueous One Solution cell proliferation assay (Promega) as previously described by Yang et al. (31). The experiments were repeated three times.

### RNA interference (RNAi).

Small interfering RNA (siRNA) used in the RNAi assay was chemically synthesized by GenePharma (China). The knockdown of endogenous RIG-I in PK-15 cells was carried out using transfection of RIG-I siRNA. Nontargeting siRNA (NC siRNA) was used as a negative control. The transfection of siRNA was performed as described previously using Lipofectamine 2000 (32). The target sequence for porcine RIG-I was 5′-GGTACAAAGTTGCAGGTAT-3′.

### Reporter gene assays.

HEK293T cells (10^5^) were seeded in 24-well plates. Lipofectamine 3000 (Invitrogen) was used as the transfection reagent; the empty Flag vector was used in the transfection process to ensure that the same amount of cells received the same amount of total plasmids. The pRL-TK Renilla luciferase reporter plasmid was used in the reporter assay to normalize the transfection efficiency. Where indicated, the HEK293T cells were mock infected or infected with SeV (100 hemagglutinating activity units [HAU]/ml) at 24 hpt. Detailed protocols have been described previously (27). The cells were lysed at 16 hpi, and the dual-specific luciferase assay kit (Promega) was used to analyze the firefly and Renilla luciferase activities according to the manufacturer's instructions. The data of the results represent the means and standard deviations of data from three independent experiments.

## RESULTS

### FMDV infection decreases RIG-I protein expression.

**FIG 1:**
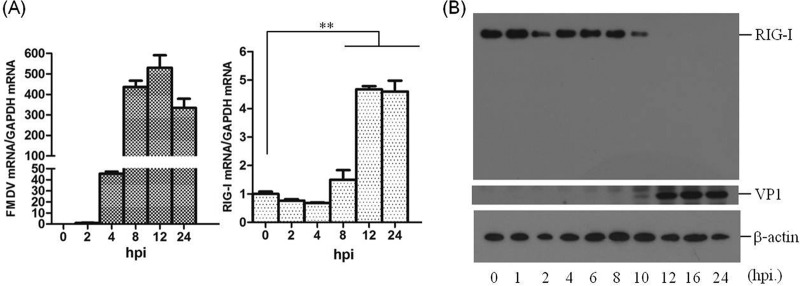
FMDV triggers RIG-I mRNA expression and decreases RIG-I protein abundance during viral infection. (A) PK-15 cells (5 × 10^5^ cells in each well) were grown in 35-mm culture dishes, the monolayers were infected with FMDV at an MOI of 0.5, and viral RNA (left panel) and RIG-I mRNA (right panel) were determined by qPCR at 0, 2, 4, 8, 12, and 24 hpi. **, *P* < 0.01, considered highly significant. The data represent results from one of the triplicate experiments. (B) Expression of endogenous RIG-I and FMDV VP1 proteins at the indicated time points postinfection (0, 1, 2, 4, 6, 8, 10, 12, 16, and 24 hpi) in PK-15 cells was determined by Western blotting. RIG-I was detected by rabbit anti-RIG-I polyclonal antibody. A mouse anti-VP1 polyclonal antibody prepared by our laboratory (unpublished data) was used to confirm the expression of viral protein, and mouse anti-β-actin was used to detect cellular β-actin.

The role of RIG-I in FMDV infection remains unknown. It was therefore necessary to investigate the state of RIG-I during FMDV infection. PK-15 cells were infected with FMDV at a multiplicity of infection (MOI) of 0.5, and the transcripts and protein levels of RIG-I was determined over time. FMDV type O strain O/BY/CHA/2010 and type Asia 1 isolate Asia1/HN/2006 were used. Similar results were observed with the two strains, therefore, we only present results from the O/BY/CHA/2010 strain. The mRNA level of RIG-I was gradually upregulated after FMDV infection (Fig. 1A). The state of RIG-I protein was also determined after FMDV infection, which showed that FMDV infection led to a significant loss of RIG-I. As shown in Fig. 1B, by 6 h postinfection (hpi), there were comparable amounts of RIG-I; however, by 8 hpi, the overall amounts of RIG-I began to decrease, and by 12 hpi, the amount of RIG-I almost could not be observed by Western blotting. These results indicated that FMDV infection can induce RIG-I mRNA expression, while RIG-I protein is gradually downregulated as infection progresses.

### RIG-I inhibits FMDV replication during virus infection.

**FIG 2:**
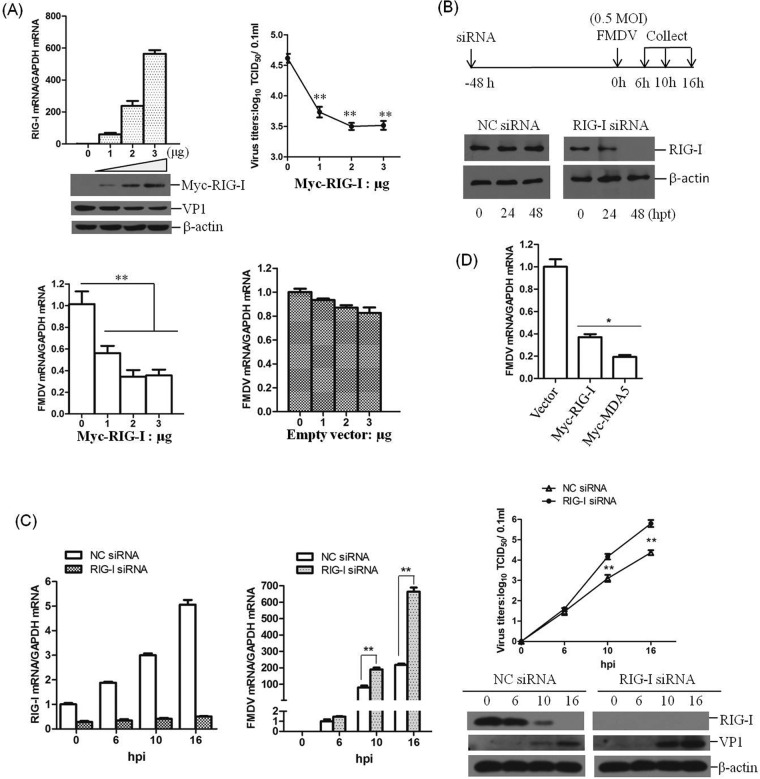
RIG-I inhibits FMDV replication during virus infection. (A) PK-15 cells (5 × 10^5^ cells in each well) were transfected with increasing amounts of Myc-tagged RIG-I-expressing plasmids (0, 1, 2, or 3 μg), and the empty vector was used in the transfection process to ensure that the cells received the same amounts of total DNA plasmids. At 12 hpt, the cells were infected with equal amounts of FMDV (MOI of 0.5) for 16 h. The expression of RIG-I mRNA and viral RNA was examined by qPCR, and expression of Myc-RIG-I and FMDV VP1 protein was examined by Western blotting. The viral titers were determined by TCID_50_ assay. The effect of empty vector DNA on the viral replication was also evaluated by the transfection of 0, 1, 2, or 3 μg of empty vector in PK-15 cells. At 12 hpt, the cells were infected with equal amounts of FMDV (MOI of 0.5) for 16 h. The expression of viral RNA was determined by qPCR. (B) Schematics of the strategy used in the RNAi assay (upper panel) and confirmation of the efficiencies of a nontargeting siRNA (NC siRNA) and RIG-I siRNA in silencing RIG-I expression (lower panel). PK-15 cells (5 × 10^5^ cells in each well) were transfected with 150 nM NC siRNA or RIG-I siRNA, and the expression of RIG-I was determined by Western blotting at 0, 24, or 48 hpt. (C) PK-15 cells (5 × 10^5^ cells in each well) were transfected with 150 nM NC siRNA or RIG-I siRNA for 48 h, followed by infection with equal amounts of FMDV (MOI of 0.5) for 0, 6, 10, and 16 h. The expression of RIG-I mRNA and viral RNA was determined by qPCR (left panel). The expression of RIG-I and viral VP1 proteins was detected by Western blotting, and viral titers were determined by TCID_50_ assay (right panel). (D) PK-15 cells were transfected with 2 μg of empty vector, Myc-RIG-I, or Myc-MDA5 plasmids. At 12 hpt, the cells were infected with equal amounts of FMDV (MOI of 0.5) for 16 h. The viral RNA levels were determined by qPCR. All of the experiments were repeated three times, with similar results. The data represent results from one of the triplicate experiments. *, *P* < 0.05, considered significant; **, *P* < 0.01, considered highly significant.

Significant upregulation of RIG-I transcripts and obvious decrease of RIG-I proteins implied a potential role of RIG-I during FMDV infection. To determine whether RIG-I is involved in FMDV replication, gain-of-function and loss-of-function assays were performed. PK-15 cells were transfected with different doses of Myc-RIG-I plasmids; at 12 hpt, the cells were infected with equal amounts of FMDV for 16 h. The viral RNA levels, viral protein abundance, and viral titers were determined and compared. As shown in Fig. 2A, the overexpression of RIG-I significantly suppressed FMDV infection. qPCR, Western blot, and viral titer analysis showed that, with increasing expression of RIG-I, FMDV replication was inhibited in a dose-dependent manner. The replication status of FMDV in RIG-I downregulated cells was also evaluated. RIG-I in PK-15 cells was knocked down using RNAi. The cells were transfected with negative control (NC) siRNA or RIG-I siRNA for 48 h and then infected with equal amounts of FMDV (MOI of 0.5) (Fig. 2B). The siRNA knockdown efficiency was confirmed by Western blotting in PK-15 cells, as shown in Fig. 2B. Viral RNA, proteins and titers in the RIG-I siRNA cells were analyzed and compared to the NC siRNA cells at the indicated time points after virus infection. The results demonstrated that FMDV replication was significantly enhanced in RIG-I siRNA cells. The levels of viral RNA and proteins and the virus titers were higher in RIG-I siRNA cells at 10 and 16 hpi (Fig. 2C). It seems that RIG-I was essential for the inhibition of FMDV infection; therefore, the virus decreases RIG-I abundance to antagonize its antiviral effect. A comparison of the effect of the overexpression of RIG-I and MDA5 on FMDV replication has also been performed. PK-15 cells were transfected with equal amounts of vector and Myc-RIG-I or Myc-MDA5 plasmids; at 12 hpt, the cells were infected with equal amounts of FMDV for 16 h. The viral RNA levels were determined and compared. Both RIG-I and MDA5 showed significant antiviral activity against FMDV, and MDA5 showed a stronger antiviral effect than RIG-I (Fig. 2D). This indicated that both MDA5 and RIG-I were involved in the host antiviral response against FMDV.

**FIG 3:**
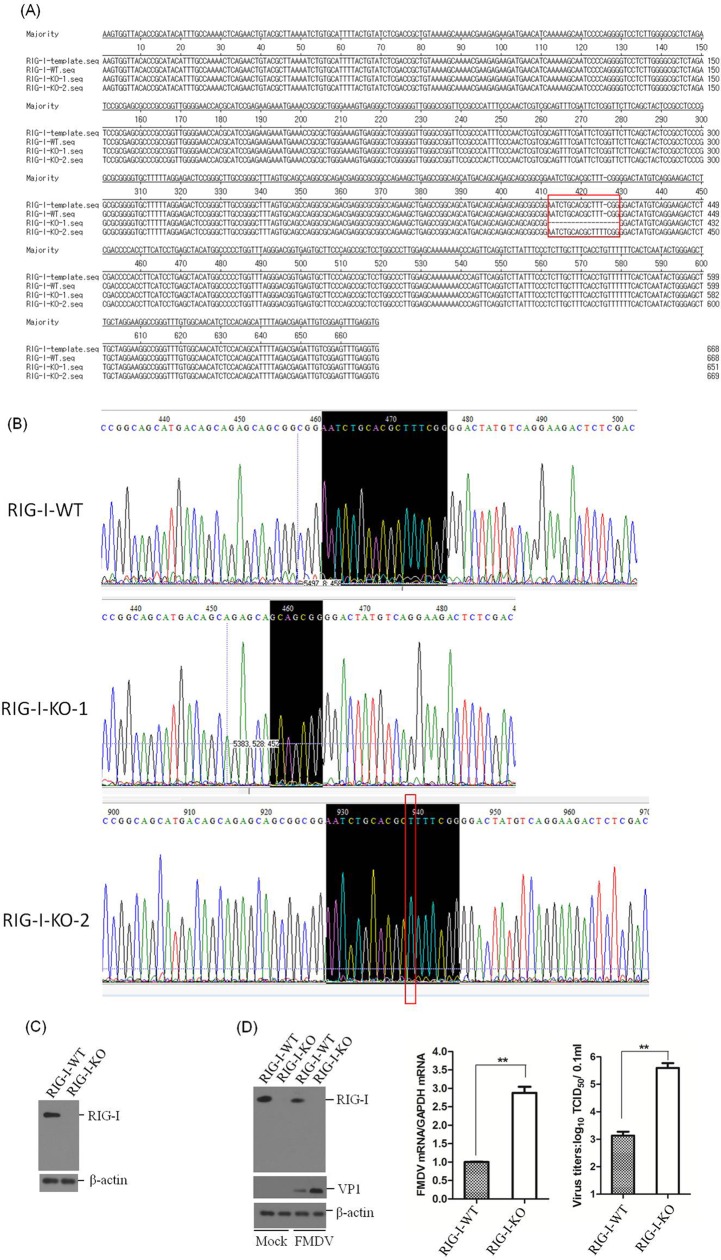
Confirmation of the establishment of RIG-I knockout PK-15 cells. (A) Alignment of the RIG-I genomic nucleotide sequence of the published RIG-I reference sequence and of the RIG-I-WT, RIG-I-KO-1, and RIG-I-KO-2 sequences using LaserGene software. The red box indicates the regions that were mutated. (B) Confirmation of the genome editing by Sanger sequencing of the PCR amplicon from the RIG-I genome of the cell lines. (C) The established RIG-I-WT and RIG-I-KO cells were seeded in six-well culture plates (5 × 10^5^ cells in each well) and grown for 24 h, and the expression of RIG-I was detected by Western blotting. (D) RIG-I-WT and RIG-I-KO PK-15 cells were seeded in six-well culture plates (5 × 10^5^ cells in each well), and the monolayer cells were mock infected or infected with 0.5 MOI of FMDV for 10 h. The expression of RIG-I and viral VP1 proteins was detected by Western blotting. The expression of viral RNA was determined by qPCR, and viral titers were determined by TCID_50_ assay. All the experiments were repeated three times with similar results. The data represent results from one of the triplicate experiments. **, *P* < 0.01 considered highly significant.

RIG-I knockout (RIG-I-KO) PK-15 cells were established by using the clustered regularly interspaced short palindromic repeats (CRISPR)/Cas9 system to evaluate the suppressive effect of RIG-I on FMDV replication. The RIG-I in the cell line was confirmed by DNA sequencing and Western blotting. The sequencing results indicated that a 17-nucleotide deletion was detected in the first exon of one allele of RIG-I (RIG-I-KO-1), and one nucleotide insertion was introduced into the other allele of RIG-I (RIG-I-KO-2) (Fig. 3A and B). The Western blot analysis demonstrated the successful knockout of RIG-I in the established cell line (Fig. 3C). One RIG-I WT cell line (RIG-I-WT) was also obtained in parallel and used as a control. Equal amounts of FMDV (MOI of 0.5) were incubated with RIG-I-WT and RIG-I-KO cells, and the viral RNA levels, the viral protein abundance, and the viral titers were determined and compared at 10 hpi. The expression of viral protein and RNA, as well as the viral titers, were significantly higher in the RIG-I-KO cells compared to the RIG-I-WT cells (Fig. 3D). This conclusively demonstrates the important antiviral role for RIG-I in FMDV infection.

### FMDV 2B protein decreases exogenous RIG-I expression.

**FIG 4:**
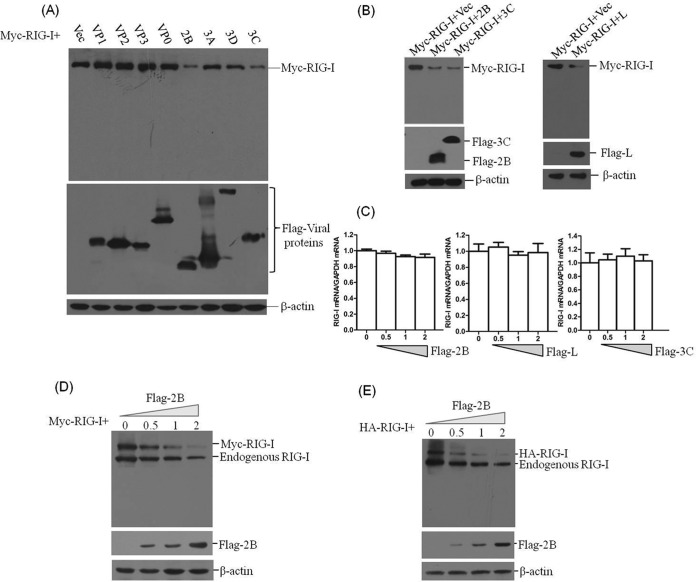

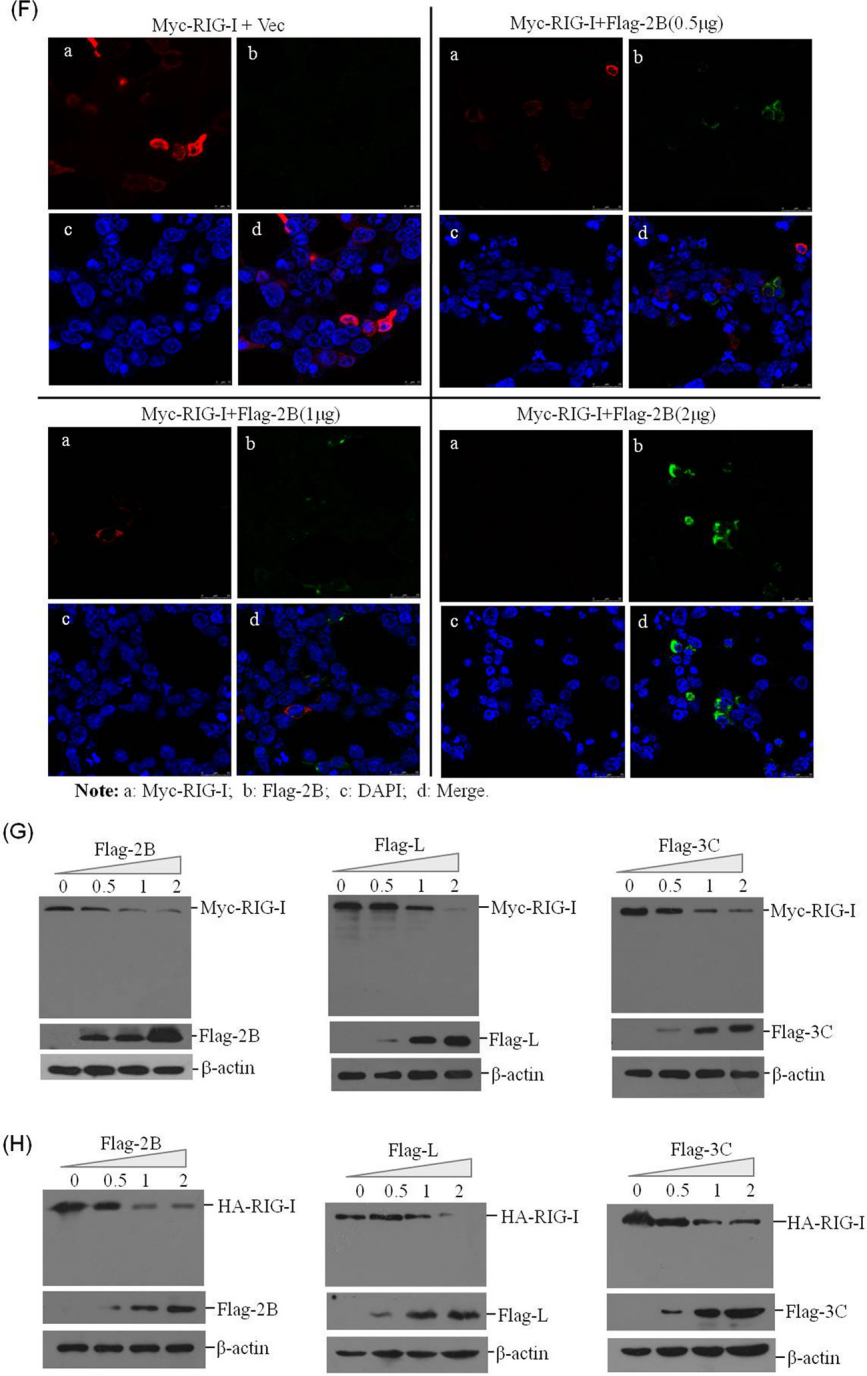
2B protein induces the reduction of exogenous RIG-I expression. (A) HEK293T cells (5 × 10^5^ cells in each well) were transfected with Myc-tagged RIG-I-expressing plasmid (2 μg), along with various plasmids expressing Flag-tagged viral proteins (VP1, VP2, VP3, VP0, 2B, 3A, 3D^pol^, or 3C^pro^) or empty Flag vector plasmid (2 μg). The expression of Myc-RIG-I and Flag-tagged viral protein was detected by Western blotting at 36 hpt. (B) HEK293T cells (5 × 10^5^ cells in each well) were transfected with Myc-tagged RIG-I-expressing plasmid (2 μg), along with various plasmids expressing Flag-tagged viral proteins (2B, 3C^pro^, or L^pro^) or empty Flag vector (2 μg). The expression of Myc-RIG-I and Flag-tagged viral protein was detected by Western blotting at 36 hpt. (C) HEK293T cells (5 × 10^5^ cells in each well) were transfected with Myc-RIG-I-expressing plasmid (2 μg), along with increasing quantities of Flag-2B-, Flag-3C-, or Flag-L-expressing plasmids (0, 0.5, 1, or 2 μg). The empty vector was used in the transfection process to ensure that the cells received the same amounts of total plasmids. The expression of RIG-I mRNA was determined by qPCR analysis at 36 hpt. (D) HEK293T cells (5 × 10^5^ cells in each well) were transfected with Myc-RIG-I-expressing plasmid (2 μg), along with increasing quantities of Flag-2B-expressing plasmid (0, 0.5, 1, or 2 μg). The expression of Myc-RIG-I and Flag-2B was detected by Western blotting with anti-RIG-I and anti-Flag antibodies at 36 hpt. (E) HEK293T cells (5 × 10^5^ cells in each well) were transfected with HA-RIG-I-expressing plasmid (2 μg), along with increasing quantities of Flag-2B-expressing plasmid (0, 0.5, 1, or 2 μg). The expression of HA-RIG-I and Flag-2B was detected by Western blotting with anti-RIG-I and anti-Flag antibodies at 36 hpt. (F) HEK293T cells (3 × 10^5^ cells in each well) were transfected with Myc-RIG-I-expressing plasmid (2 μg), along with increasing quantities of Flag-2B-expressing plasmids (0, 0.5, 1, or 2 μg). The expression of Myc-RIG-I and Flag-2B was detected by IFA analysis at 36 hpt. Cells were double immunostained for Myc-RIG-I (red) and Flag-2B (green); cellular nuclei were counterstained with DAPI (blue). (G) HEK293T cells (5 × 10^5^ cells in each well) were transfected with Myc-RIG-I-expressing plasmid (2 μg), along with increasing quantities of Flag-2B-, Flag-L-, or Flag-3C-expressing plasmid (0, 0.5, 1, or 2 μg). The expression of Myc-RIG-I and Flag-tagged viral proteins was detected by Western blotting with anti-Myc and anti-Flag antibodies at 36 hpt. (H) HEK293T cells (5 × 10^5^ cells in each well) were transfected with HA-RIG-I-expressing plasmid (2 μg), along with increasing quantities of Flag-2B-, Flag-L-, or Flag-3C-expressing plasmids (0, 0.5, 1, or 2 μg). The expression of HA-RIG-I and Flag-tagged viral proteins was detected by Western blotting with anti-HA and anti-Flag antibodies at 36 hpt.

FMDV infection contributes to reduction of RIG-I. To identify the viral proteins that are responsible for the loss of RIG-I protein expression, plasmids encoding Flag-tagged viral proteins and Myc-tagged RIG-I were cotransfected into HEK293T cells. After 36 h, the abundance of Myc-RIG-I was detected by Western blotting. It was observed that FMDV 2B, 3C^pro^, and L^pro^ significantly decreased expression of RIG-I (Fig. 4A and B). To investigate whether the decrease in RIG-I was the result of the specific decrease in mRNA expression, the abundance of RIG-I mRNA in Myc-RIG-I- and Flag-2B-, Flag-3C-, or Flag-L-cotransfected cells was measured by qPCR. There was no significant decrease in RIG-I mRNA levels (Fig. 4C). This implied that FMDV 3C^pro^ and L^pro^ degraded RIG-I or that 3C^pro^- and L^pro^-induced cleavage of host eIF4G limited the synthesis of RIG-I. However, the decrease in RIG-I induced by 2B protein revealed a novel mechanism evolved by FMDV to antagonize the host antiviral response. To further determine the effect of 2B on RIG-I protein expression, dose-response experiments were carried out. Plasmids encoding Myc-RIG-I with a carboxyl-terminal Myc tag and HA-RIG-I with an amino-terminal HA tag were both used for this analysis to confirm 2B-induced reduction of RIG-I. It was observed that both exogenous and endogenous RIG-I protein levels were reduced by expression of 2B, showing a dose-dependent manner. However, no cleaved bands were observed using the anti-RIG-I antibody (Fig. 4D and E). An indirect immunofluorescence assay (IFA) was subsequently performed, which also indicated the dose-dependent effect (Fig. 4F). We also compared the 2B-, L^pro^-, and 3C^pro^-induced reduction of RIG-I. Plasmids encoding Flag-tagged 2B, L^pro^, or 3C^pro^ proteins and Myc- or HA-tagged RIG-I were cotransfected into HEK293T cells. After 36 h, the expression of Myc-RIG-I or HA-RIG-I was detected by Western blotting. This suggested that 2B, L^pro^, and 3C^pro^ showed approximately similar inductive abilities for reducing RIG-I protein levels (Fig. 4G and H). These results indicate that FMDV 2B can abrogate RIG-I protein expression.

### FMDV 2B protein decreases endogenous RIG-I and impairs RIG-I-mediated signaling.

**FIG 5:**
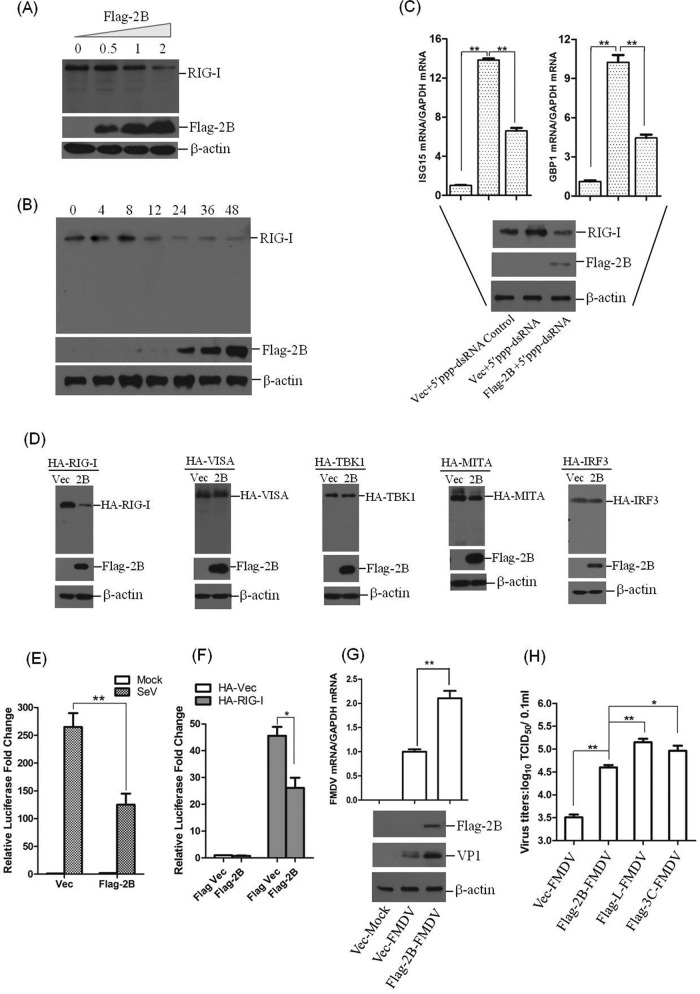
2B protein induces the reduction of RIG-I and suppresses RIG-I-mediated signal transduction. (A) PK-15 cells (5 × 10^5^ cells in each well) were grown in six-well plates, and the monolayer cells were transfected with different doses of Flag-2B-expressing plasmids (0, 0.5, 1, or 2 μg). The expression of endogenous RIG-I and Flag-2B proteins was detected by Western blotting at 48 hpt. RIG-I was detected by using rabbit anti-RIG-I polyclonal antibody. (B) PK-15 cells (5 × 10^5^ cells in each well) were transfected with Flag-2B-expressing plasmid (2 μg). The cells were collected at 0, 4, 8, 12, 24, 36, and 48 hpt, and the cell lysates were analyzed by Western blotting to detect the expression levels of RIG-I and any possible cleaved bands. (C) PK-15 cells (5 × 10^5^ cells in each well) were transfected with Flag-2B-expressing plasmid (2 μg) or empty vector (2 μg) in the presence or absence of 5′ppp-dsRNA control or 5′ppp-dsRNA (1 μg/ml; InvivoGen). The expression of two ISGs (ISG15 and GBP1) was determined by qPCR assay at 24 hpt. The data represent results from one of the triplicate experiments. (D) HEK293T cells (5 × 10^5^ cells in each well) were transfected with HA-RIG-I-, HA-VISA-, HA-TBK1-, HA-MITA-, or HA-IRF3-expressing plasmids (2 μg), along with Flag-2B-expressing plasmid or empty vector (2 μg). The expression of HA-RIG-I, HA-VISA, HA-TBK1, HA-MITA, HA-IRF3, and Flag-2B was detected by Western blotting at 48 hpt. Mouse anti-HA antibody was used to detect HA-tagged proteins. (E) HEK293T cells (10^5^ cells in each well) were seeded in 24-well plates, and the monolayer cells were transfected with Flag-2B-expressing plasmid (0.1 μg) or empty vector (0.1 μg), along with IFN-β luciferase reporter plasmid (0.05 μg). pRL-TK Renilla luciferase reporter plasmid (0.01 μg) was used in the reporter assay to normalize the transfection efficiency. At 24 h after transfection, the cells were left infected or uninfected with SeV (100 HAU/ml) for 16 h. A dual-specific luciferase assay kit was used to analyze the luciferase activities of firefly and Renilla. Empty vector plasmid was used in the transfection process to ensure that the cells received the same amounts of total plasmids. The data represent the means and standard deviations from three independent experiments. (F) HEK293T cells (10^5^ cells in each well) were cotransfected with HA-RIG-I-expressing plasmid (0.1 μg) or empty vector (0.1 μg) and Flag-2B-expressing plasmid (0.1 μg) or empty Flag vector (0.1 μg), along with IFN-β luciferase reporter plasmid (0.1 μg). The pRL-TK Renilla luciferase reporter plasmid (0.01 μg) was used in the reporter assay to normalize the transfection efficiency. The dual-specific luciferase assay kit was used to analyze the luciferase activities of firefly and Renilla at 24 hpt as described for panel E. (G) FMDV 2B enhances virus replication in infected cells. PK-15 cells (5 × 10^5^ cells in each well) were transfected with Flag-2B-expressing plasmid (2 μg) or empty vector (2 μg). At 24 h after transfection, the cells were infected or uninfected with FMDV (MOI of 0.5) for 12 h. The expression of viral RNA was determined by qPCR assay. The viral VP1 proteins were detected by Western blotting. (H) FMDV 2B, L^pro^, and 3C^pro^ enhance virus replication in infected cells. PK-15 cells (5 × 10^5^ cells in each well) were transfected with empty vector, Flag-2B-, Flag-3C-, or Flag-L-expressing plasmids (2 μg). At 24 h after transfection, the cells were infected with FMDV (MOI of 0.5) for 12 h. The viral titers were determined by using a TCID_50_ assay. All of the above-described experiments were repeated three times, with similar results. **, *P* < 0.01, considered highly significant.

FMDV 2B-induced reduction of endogenous RIG-I was further examined by a Western blot analysis. PK-15 cells were transfected with increasing amounts of Flag-2B-expressing plasmids, and the cells were collected at 48 hpt for analysis of the RIG-I protein abundance. The results were consistent with those noted above, since 2B decreased RIG-I protein levels in a dose-dependent manner (Fig. 5A). The kinetics of the 2B-induced decrease of RIG-I were subsequently examined to detect any RIG-I cleavage product(s). PK-15 cells were transfected with Flag-2B-expressing plasmid. The cells were then collected at different time points, and the expression of RIG-I was determined by Western blotting. As shown in Fig. 5B, 2B gradually induced the reduction of RIG-I as time progressed; however, no cleavage bands were observed.

The suppressive activity of 2B on RIG-I-mediated signal transduction was also explored to confirm the 2B-induced reduction of RIG-I. A synthetic analog of viral double-stranded DNA (dsRNA), 5′ppp-dsRNA, a potent activator of RIG-I, was used to activate RIG-I-mediated signal transduction (33). PK-15 cells were transfected with 5′ppp-dsRNA accompanied by the empty vector or Flag-2B-expressing plasmid, respectively. The expression of IFN-stimulated gene 15 (ISG15) and IFN-induced guanylate-binding protein 1 (GBP1) was determined by qPCR. The 5′ppp-dsRNA-induced upregulation of ISG15 and GBP1 was significantly suppressed (Fig. 5C). This result implies that 2B decreases RIG-I, resulting in impaired RIG-I-mediated signal transduction. To investigate whether 2B decreases other adaptor molecules in the RIG-I-mediated signaling pathway, HA-tagged RIG-I-, VISA-, TBK1-, mediator of IRF3 activation (MITA)-, or IRF3-expressing plasmids were cotransfected with empty vector or Flag-2B-expressing plasmid, respectively. The expression level of each protein was detected by Western blotting. Compared to the empty vector plasmid control, FMDV 2B significantly induces the reduction of RIG-I, whereas 2B did not induce the reduction of VISA, TBK1, MITA, and IRF3 (Fig. 5D). SeV is a model virus that has been widely used to investigate RIG-I-mediated signaling in cell culture due to its significant inductive activity to activate the type I IFN pathway. Therefore, we used SeV to analyze the affection of 2B on RIG-I-mediated signaling. The effects of 2B on SeV-triggered or RIG-I-mediated promoter activation of IFN-β were also determined by reporter assays (25). HEK293T cells were transfected with Flag-2B-expressing plasmid or empty vector, along with IFN-β luciferase reporter plasmid and pRL-TK Renilla luciferase reporter plasmid. The SeV- and RIG-I-induced promoter activation of IFN-β was determined. The results showed that 2B inhibited the activation of IFN-β promoters in HEK293T cells (Fig. 5E and F).

As described above, RIG-I inhibits FMDV replication in host cells. Here, we observed the 2B-induced reduction of RIG-I and suppression of RIG-I-mediated antiviral signal transduction. To determine whether 2B affected the replication of FMDV, PK-15 cells were transfected with empty vector or Flag-2B-expressing plasmid for 24 h, and then the cells were mock infected or infected with an equal amount of FMDV. The expression levels of viral RNA and viral protein was examined at 12 hpi. The overexpression of 2B significantly enhanced FMDV replication in PK-15 cells (Fig. 5G). The effects on infectivity of overexpression of 2B, L^pro^, and 3C^pro^ were also determined for comparison purposes. The empty vector or plasmids encoding Flag-tagged 2B, L^pro^, or 3C^pro^ proteins were transfected into PK-15 cells. After 24 h, the cells were infected with an equal amount of FMDV. Viral titers were determined by TCID_50_ assay at 12 hpi. The results suggested that the overexpression of 2B, L^pro^, and 3C^pro^ significantly promoted FMDV replication and that L^pro^ and 3C^pro^ showed stronger ability than 2B (Fig. 5H). These results indicate that FMDV 2B, L^pro^, and 3C^pro^ proteins significantly promote viral replication during FMDV infection.

### FMDV 2B does not induce the cleavage of eIF4GI and cellular apoptosis in PK-15 cells.

**FIG 6:**
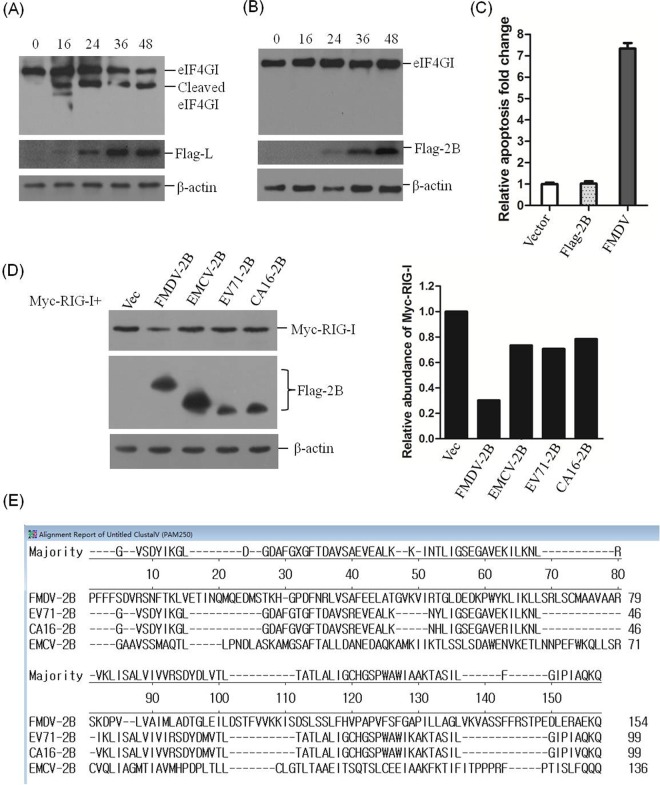
2B does not induce cleavage of eIF4GI and cellular apoptosis. (A) PK-15 cells (5 × 10^5^ cells in each well) were transfected with 2 μg of Flag-L-expressing plasmid, and the cells were collected at 0, 16, 24, 36, and 48 hpt. The protein levels of eIF4GI were determined by Western blotting with anti-eIF4GI polyclonal antibody produced in rabbits, and Flag-L protein was detected by using anti-Flag antibody. (B) PK-15 cells were transfected with Flag-2B-expressing plasmid, as described for panel A, and the protein levels of eIF4GI were determined by Western blotting. (C) PK-15 cells (5 × 10^5^ cells in each well) were grown in each well of six-well plates. The monolayer cells were transfected with 2 μg of empty vector or 2 μg of Flag-2B-expressing plasmid using Lipofectamine 2000. Another well of PK-15 cells was infected with FMDV at an MOI of 0.05 as a positive control of apoptosis. The apoptosis status of the transfected and infected cells was analyzed by AnnV-PI staining and flow cytometric analysis at 24 hpt or hpi. The relative fold change in apoptosis of vector-transfected, Flag-2B-transfected, and virus-infected cells was determined. The experiments were repeated three times with similar results. (D) HEK293T cells (5 × 10^5^ cells in each well) were transfected with Myc-tagged RIG-I-expressing plasmid (2 μg), along with FMDV, EMCV, EV71, or CA16 Flag-2B-expressing plasmids (2 μg). The expression of Myc-RIG-I and Flag-tagged 2B proteins was detected by Western blotting at 36 hpt (left panel). The change in the abundance of Myc-RIG-I in the transfectants was determined by densitometric analysis using ImageJ software and normalized to β-actin (right panel). (E) Amino acid alignment of the 2B coding sequences of sequenced FMDV, EMCV, EV71, and CA16 genomes using LaserGene software (DNASTAR, Inc.). The GenBank accession numbers of the viral 2B sequences are AET43040.1 (FMDV), AFU64561.1 (EMCV), NP_740531 (EV71), and ALB74859.1 (CA16).

FMDV infection causes a rapid shutdown of cellular protein synthesis, and it is widely known that L^pro^ can induce the cleavage of host eIF4G, which limits the synthesis of host proteins (34, 35). The 2B protein decreased RIG-I expression in PK-15 cells, and no cleaved bands were observed. To determine whether 2B limited the synthesis of RIG-I by inducing the cleavage of eIF4G, PK-15 cells were transfected with Flag-2B- or Flag-L-expressing plasmids, and the kinetics of major eIF4G isoform (eIF4GI) protein levels were examined. L^pro^ cleaved eIF4GI as previously reported (36) (Fig. 6A); however, the expression of 2B did not affect eIF4GI protein levels, indicating that 2B did not induce the cleavage of eIF4G in PK-15 cells (Fig. 6B). To further determine whether the 2B protein induces cellular apoptosis in PK-15 cells, which may result in the disappearance of RIG-I, we analyzed the apoptosis levels of the cells after transfecting cells with control Flag vector or Flag-2B-expressing plasmid. FMDV infection-induced apoptosis was used as a positive control. The results showed that 2B expression did not induce significant apoptosis compared to the vector-transfected PK-15 cells, but FMDV infection induced significant apoptosis (Fig. 6C). Collectively, these results indicate that 2B induces the reduction of RIG-I independent of the cleavage of eIF4G or the induction of cellular apoptosis.

We also investigated whether other picornavirus 2B proteins possess the ability to induce the reduction of RIG-I. Encephalomyocarditis virus (EMCV), enterovirus 71 (EV71), and coxsackievirus A16 (CA16) 2B proteins were used to explore similar implications. HEK293T cells were seeded in six-well plates, and Myc-RIG-I-expressing plasmid and plasmids expressing Flag-tagged FMDV, EMCV, EV71, or CA16 2B were cotransfected into the monolayer cells using Lipofectamine 2000. The lysates were analyzed using anti-Myc and anti-Flag antibodies. The results showed that only FMDV 2B induced significant reduction of RIG-I, whereas EMCV, EV71, and CA16 2B proteins did not affect RIG-I expression (Fig. 6D). The amino acid sequences of the 2B proteins of FMDV, EMCV, EV71, and CA16 were also compared, and FMDV 2B sequence showed extremely low sequence identities with these other 2B sequences, findings similar to those of a previous study (4) (Fig. 6E). Therefore, not all 2B proteins of picornaviruses have the ability to induce reduction of RIG-I, since only the FMDV 2B protein was determined to possess this activity.

### Impact of proteasome, lysosome, and caspase inhibitors on FMDV- and 2B-induced reduction of RIG-I, as well as the functional region of 2B responsible for this effect on RIG-I.

**FIG 7:**
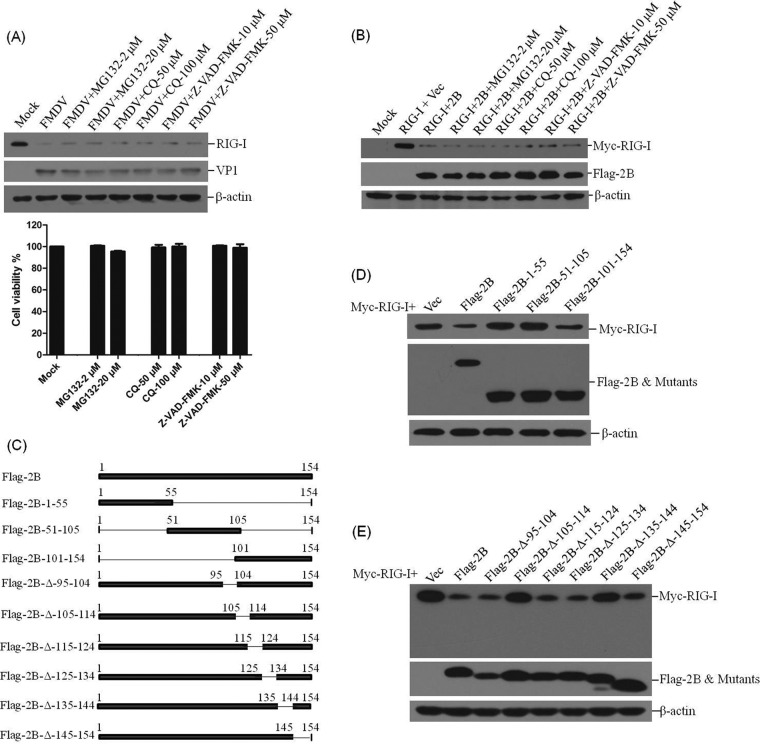
Effect of MG132, CQ, and Z-VAD-FMK on FMDV- and 2B-induced reduction of RIG-I, and the regions of 2B responsible for the activity. (A) PK-15 cells (5 × 10^5^ cells in each well) were mock infected or infected with FMDV (MOI of 0.5) and maintained in the presence or absence of the proteasome inhibitor MG132 (2 or 20 μM), the lysosome inhibitor CQ (50 or 100 μM), or the caspase inhibitor Z-VAD-FMK (10 or 50 μM) for 12 h. The expression of endogenous RIG-I and viral VP1 proteins was detected by Western blotting (upper panel). The cytotoxicity of MG132, CQ, and Z-VAD-FMK on PK-15 cells were also determined by MTS assay (lower panel). (B) HEK293T cells (5 × 10^5^ cells in each well) were cotransfected with Myc-RIG-I-expressing plasmid (2 μg) and empty vector (2 μg) or Flag-2B-expressing plasmid (2 μg) and maintained in the presence or absence of MG132 (2 or 20 μM), CQ (50 or 100 μM), or Z-VAD-FMK (10 or 50 μM) for 36 h. The expression of Myc-RIG-I and Flag-2B proteins was detected by Western blotting. (C) Schematics of a series of Flag-tagged truncated 2B constructs. (D) HEK293T cells (5 × 10^5^ cells in each well) were cotransfected with Myc-RIG-I-expressing plasmid (2 μg) and Flag-2B-expressing plasmid (2 μg), empty vector, or indicated 2B mutant-expressing plasmids. The expression of Myc-RIG-I and Flag-tagged proteins was detected at 36 hpt by Western blotting. (E) HEK293T cells (5 × 10^5^ cells in each well) were cotransfected with Myc-RIG-I-expressing plasmid (2 μg) and Flag-2B-expressing plasmid (2 μg), empty vector, or indicated 2B mutant-expressing plasmids. The expression of Myc-RIG-I and Flag-tagged proteins was detected at 48 hpt by Western blotting.

Poliovirus-induced cleavage of RIG-I occurs by a process independent of proteasomes and lysosomes (19). To determine whether the proteasomes, lysosomes, or caspase-dependent pathways play roles in FMDV- or 2B-induced reduction of RIG-I, the proteasome inhibitor MG132, the lysosome inhibitor CQ, and the general caspase inhibitor Z-VAD-FMK were used to evaluate the inhibitive effects. PK-15 cells were infected with FMDV and maintained in the presence or absence of the inhibitors. The expression of RIG-I at 12 hpi was detected by Western blotting. In the presence of MG132, CQ, or Z-VAD-FMK, FMDV infection-induced reduction of RIG-I was not suppressed (Fig. 7A). The cytotoxicity of the used inhibitors against PK-15 cells was determined by MTS assay. All doses of the inhibitor used in the experiments showed no detectable cell death (Fig. 7A). The effects of MG132, CQ, or Z-VAD-FMK on 2B-induced reduction of RIG-I were also examined. Flag-2B- and Myc-RIG-I-expressing plasmids were cotransfected into HEK293T cells, and the cells were cultured with or without the inhibitors. The expression of RIG-I was detected at 36 hpt by Western blotting. No inhibitory effects of MG132, CQ, or Z-VAD-FMK on the reduction of RIG-I were observed (Fig. 7B). These results suggest that the reduction of RIG-I during FMDV infection is independent of proteasome, lysosome, and caspase pathways. In addition, 2B-induced reduction of RIG-I is also independent of proteasome, lysosome, and caspase pathways. Whether 2B has any proteinase activity was predicted using Interpro (http://www.ebi.ac.uk/interpro/scan.html) and Pfam (http://pfam.xfam.org/search) functional analysis tools. The results suggested that 2B does not contain any potential proteolytic domains (data not shown).

To determine further the functional domain of 2B that is responsible for reduction of RIG-I, a series of truncation mutants of Flag-2B-expressing plasmids were generated through PCR-based site-directed mutagenesis (Fig. 7C). The Myc-RIG-I-expressing plasmid was cotransfected with various plasmids expressing truncated forms of 2B, and the abundance of RIG-I was detected at 36 hpt by Western blotting. Two truncated mutants that included the regions from amino acids 1 to 55 and amino acids 51 to 105 of 2B did not induce the reduction of RIG-I, and a mutant that included the carboxyl-terminal 101- to 154-amino-acid region of 2B was observed to induce the reduction of RIG-I (Fig. 7D), whereas the carboxyl-terminal 101- to 154-amino-acid region showed lower activity compared to the complete 2B protein. The functional domain in the carboxyl-terminal 101- to 154-amino-acid region was subsequently analyzed; a series of plasmids expressing carboxyl-terminal truncated mutants of Flag-2B was cotransfected with Myc-RIG-I-expressing plasmid, and the expression of Myc-RIG-I was detected by Western blotting. The results indicated that deletion of the carboxyl-terminal 105- to 114-amino-acid or 135- to 144-amino-acid region in 2B abrogated the reduction of RIG-I (Fig. 7E). This suggests that the carboxyl-terminal 105- to 114-amino-acid and 135- to 144-amino-acid regions are essential for inducing the reduction of RIG-I.

### 2B interacts with RIG-I, and the interaction is essential for inhibition of RIG-I protein expression.

**FIG 8:**
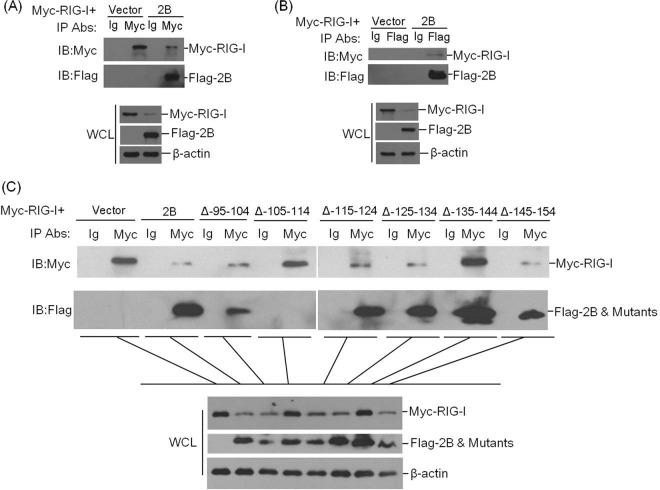
2B interacts with RIG-I. (A) 2 × 10^6^ HEK-293T cells were seeded in a 10-cm dish. The monolayer cells were cotransfected with 8 μg of Myc-RIG-I-expressing plasmid and 5 μg of empty Flag vector or 5 μg of Flag-2B-expressing plasmid. The cells were lysed at 24 hpt, and the lysates were immunoprecipitated with mouse anti-Myc or mouse normal IgG antibody and subjected to Western blotting. Whole-cell lysates (WCL) and immunoprecipitation (IP) antibody-antigen complexes were analyzed by immunoblotting (IB) using anti-Flag, anti-Myc, or anti-β-actin antibodies. (B) Similar transfection and immunoprecipitation experiments were performed as described above for panel A. However, the lysates were immunoprecipitated with rabbit anti-Flag antibody or rabbit normal IgG antibody and then subjected to Western blotting with anti-Myc or anti-Flag antibodies. (C) 2 × 10^6^ HEK293T cells were seeded in a 10-cm dish. The monolayer cells were cotransfected with Myc-RIG-I-expressing plasmid (8 μg) and empty Flag vector (5 μg), Flag-2B-expressing plasmid (5 μg), or a series of constructs expressing Flag-tagged truncated 2B (5 μg). The cells were lysed at 24 hpt, and the lysates were immunoprecipitated with mouse anti-Myc antibody or mouse normal IgG antibody and subjected to Western blotting. WCL and IP antibody-antigen complexes were analyzed by immunoblotting (IB) with anti-Flag, anti-Myc, or anti-β-actin antibodies.

To investigate a possible interaction between 2B protein and RIG-I, HEK293T cells were cotransfected with Myc-RIG-I-expressing plasmid and empty vector or Flag-2B-expressing plasmid. The lysates were immunoprecipitated with anti-Myc antibody and analyzed by Western blotting. As shown in Fig. 8A, Myc-RIG-I pulled down Flag-2B, which indicated that 2B interacts with RIG-I. The whole-cell lysates and the immunoprecipitated Myc-RIG-I proteins were visualized by using the regular enhanced chemiluminescence detection reagents; the immunoprecipitated Flag-2B proteins were visualized by using a highly sensitive detection method (the SuperSignal West Femto maximum sensitivity substrate kit). Therefore, the intensity of the Flag-2B staining is stronger than that of Myc-RIG-I. A reverse immunoprecipitation experiment was performed with anti-Flag antibody to confirm the interaction. Similarly, it was observed that Flag-2B also immunoprecipitated Myc-RIG-I (Fig. 8B). Because the carboxyl-terminal 105- to 114-amino-acid and 135- to 144-amino-acid regions of 2B were determined to be essential for inducing the reduction of RIG-I (Fig. 7E), HEK293T cells were cotransfected with Myc-RIG-I-expressing plasmid and empty vector, Flag-2B-expressing plasmid, or a series of previously constructed plasmids expressing Flag-tagged truncated mutants of 2B. An immunoprecipitation assay was then performed to explore whether the interaction between 2B and RIG-I was associated with the 2B-induced reduction of RIG-I. The lysates were immunoprecipitated with anti-Myc antibody. As shown in Fig. 8C, 2B was coprecipitated with Myc-RIG-I. Meanwhile, it was observed that the 2B mutant with the deletion of the carboxyl-terminal 105- to 114-amino-acid region failed to interact with RIG-I. Therefore, we determined that 2B interacts with RIG-I and the carboxyl-terminal 105- to 114-amino-acid region is the interaction region that is essential for inducing the reduction of RIG-I. In addition, we observed that the 2B mutant with the deletion of the carboxyl-terminal 135- to 144-amino-acid region, which was proven to fail to trigger the 2B-induced reduction of RIG-I (Fig. 7E), also interacted with RIG-I (Fig. 8C). This implies that this region did not include the interaction sites; however, this region may include the functional domain that induces the reduction of RIG-I or recruits other proteins to degrade RIG-I. These results suggest that 2B interacts with RIG-I and that this interaction is essential for the 2B-induced reduction of RIG-I.

## DISCUSSION

RIG-I is one of the PRRs in picornaviruses (20, 21), which has an important role in host defense to viral pathogens. Despite the sensing of picornavirus RNA being predominantly performed by MDA5 (17, 18), the RNA of coxsackievirus, which belongs to the picornavirus family, also includes the RNA structures that can be recognized by RIG-I (20, 21). AU-rich sequences present in the picornavirus genome might also confer RIG-I binding and signaling (19, 21). In PK-15 cells, it has been determined that the sensing of FMDV is solely mediated by MDA5 but not RIG-I in the early infection period (37). However, whether the silence of RIG-I affects FMDV replication remains unclear. A comparison of the effect of the overexpression of RIG-I and MDA5 on FMDV growth suggested that both RIG-I and MDA5 showed significant antiviral activity against FMDV, and MDA5 showed a stronger antiviral effect than RIG-I. MDA5 could upregulate type I IFN production, and RIG-I had no significant effect on type I IFN production during FMDV infection (37). MDA5-mediated type I IFN induction may have been responsible for the stronger antiviral activity of MDA5. RIG-I may perform its antiviral activity against FMDV through other mechanisms independent of type I IFN signaling. Although MDA5 showed a stronger antiviral effect than RIG-I, the loss-of-function assays indicate that RIG-I is essential for the host to suppress FMDV replication. RIG-I can displace IAV NS1 protein and vaccinia virus E3L bound to RNA, inhibiting viral replication independent of the IFN signaling (14, 38, 39). RIG-I also binds the panhandle promoter of incoming IAV and directly suppresses viral replication by destabilizing nucleocapsids (39). In hepatitis B virus (HBV) infection, RIG-I counteracts the interaction of viral polymerase with the pregenomic RNA to suppress HBV replication (38). RIG-I is cleaved by viral proteinase 3C^pro^ in cells infected with poliovirus and EMCV (19, 21). It implies an important role of RIG-I during picornavirus infection. In this study, we found that the abundance of RIG-I was significantly decreased in response to FMDV infection. Cleavage of RIG-I has been observed in cells infected with EMCV, poliovirus, echovirus type 1, and rhinovirus (19, 21, 40). Although cleavage products were not detected in FMDV-infected cells, FMDV-induced RIG-I reduction may be partially similar to other picornavirus-induced cleavage of RIG-I.

The gain-of-function and loss-of-function experiments confirmed the antiviral role of RIG-I during FMDV infection. No matter whether RIG-I can sense FMDV RNA or has other unknown functions, it is believed to be involved in the host anti-FMDV process. EMCV replication is suppressed in RIG-I-deficient mice (22). However, knockdown or knockout of RIG-I in PK-15 cells significantly enhances FMDV replication. Picornaviridae is a large and diverse family of viruses, and perhaps RIG-I performs different roles in EMCV- and FMDV-infected cells. Whether RIG can displace FMDV viral proteins or destabilizing viral replication complexes remains unclear. However, we consider that RIG-I possibly exerts its direct antiviral function during FMDV infection. Although the precise mechanisms underlying the antiviral activity of RIG-I are not fully understood, they are generally thought to be involved in antiviral response. In this study, we show for the first time the antiviral activity of RIG-I against FMDV.

The antagonistic role of 2B in host cells was confirmed in our study. Both the 5′ppp-dsRNA-induced signal transduction and SeV-triggered promoter activation of IFN-β were significantly suppressed by overexpression of the 2B protein. Overexpression of 2B enhanced the replication of FMDV in PK-15 cells. 2B reveals various different functions in virus-infected cells. The enhancing effect of 2B expression on FMDV replication can be due to many reasons. For example, 2B-induced membrane rearrangements may improve the viral multiplication. We have demonstrated the antiviral activity of RIG-I during FMDV infection. Therefore, we deem that 2B-induced decrease of RIG-I may be also one of the factors that facilitate FMDV multiplication.

FMDV 2B is an antagonistic factor in host cells that is involved in disruption of the secretory pathway by blocking ER-to-Golgi transport (4, 5). Comparing the functions of various picornavirus 2B proteins suggests that enterovirus and rhinovirus 2B can affect calcium homeostasis and protein transport, whereas, similar to the hepatitis A virus and EMCV 2B proteins, FMDV 2B has no obvious effect on calcium homeostasis and protein transport (41, 42), which indicates that the 2B proteins of picornaviruses support virus replication in different manners. In this study, we also found that only FMDV 2B possesses the activity to induce reduction of RIG-I; EMCV, EV71, and CA16 2B proteins did not significantly induce the reduction of RIG-I (Fig. 6). An amino acid sequences alignment of FMDV, EMCV, EV71, and CA16 2B indicated a low identity between FMDV 2B sequence and those of the other three picornaviruses. This implies that different 2B proteins have different roles in cells infected with different picornaviruses.

RIG-I is degraded during EMCV, poliovirus, echovirus type 1, and rhinovirus infection in a proteasome- and lysosome-independent pathway (19). In response to FMDV infection, the proteasome-, lysosome-, and caspase-dependent pathways are not involved in reduction of RIG-I. The three viral proteins 2B, L^pro^, and 3C^pro^ contributed to the reduction of RIG-I. FMDV L^pro^ and 3C^pro^ are known as viral proteinases and cleave various host proteins (43–45), and the 3C^pro^ of other picornaviruses has been shown to cleave RIG-I in host cells (19). In this study, L^pro^ and 3C^pro^ showed stronger ability to enhance FMDV replication compared with 2B; this was possibly due to the delicate viral proteinase activity of L^pro^ and 3C^pro^. L^pro^ impairs host antiviral responses by various different mechanisms, such as cleavage of eIF4G to shut off host protein synthesis, inhibition of IFN-α/β production, and catalyzing deubiquitination of innate immune signaling molecules (46, 47). The viral proteinase activity of L^pro^ and 3C^pro^ may lead to a reduction of RIG-I during FMDV infection. L^pro^- and 3C^pro^-induced cleavage of eIF4G may also result in the reduction of RIG-I. 2B did not affect eIF4G protein levels in PK-15 cells, indicating that it does not shut off host protein synthesis (Fig. 6). 2B is not a viral proteinase, and our results suggest that 2B lacks a proteolytic domain. However, 2B significantly induced the reduction of RIG-I in cultured cells, and 2B-induced reduction of RIG-I also occurred in a proteasome-, lysosome-, and caspase-independent manner. Inducing the reduction of host protein is a novel property of FMDV 2B, although the exact mechanism remains unknown.

A recent study demonstrated that 2B is a membrane-associated protein that acts as a viroporin and that 2B can induce intense autophagy in BHK-21 cells, thereby enhancing the release of viruses (48). The multiple alignment of the 2B coding sequences showed that it is highly conserved among all seven serotypes of FMDV (48). This implies that 2B contributes to the increased virus propagation and is associated with the infection mechanism of FMDV. The viroporin activity of FMDV 2B may affect the cellular apoptosis response. However, we found that transfection of the plasmid expressing FMDV 2B protein did not induce significant apoptosis in PK-15 cells. The cytopathic effect induced by FMDV infection in PK-15 cells was not apparent and sensitive as it is in BHK cells (49, 50), and whether 2B mediates different apoptotic effects in different cells, which lead to different cytopathic effects, remains unclear. Alterations in the 2B protein that do not affect the functions of 2B to permeabilize membranes or block protein secretion can also impair coxsackie B3 virus growth, suggesting additional functions for picornavirus 2B (51). 2B-mediated interaction with host proteins has also been implied to enhance rhinovirus growth in mouse cells (52). 2B-induced reduction of host cell protein may be a new function of 2B. A direct interaction was observed between RIG-I and 2B (Fig. 8), and the interaction was deemed to be essential for 2B-induced reduction of RIG-I. This implies that the 2B protein interacts with RIG-I and then induces the reduction of RIG-I. The carboxyl-terminal 105- to 114-amino-acid and 135- to 144-amino-acid regions were indispensable for 2B-induced reduction of RIG-I. Two putative transmembrane domains in 2B from amino acids 83 to 104 and amino acids 119 to 137 have been reported to associate with its viroporin activity (41). Our results indicated that the deletion of the putative transmembrane domains in 2B did not affect 2B-induced reduction of RIG-I. The carboxyl-terminal 105- to 114-amino-acid and 135- to 144-amino-acid regions were also out of the putative transmembrane domains. It suggested that the activity of 2B to induce reduction of RIG-I is independent of its viroporin activity. The carboxyl-terminal 105- to 114-amino-acid region of 2B was essential for the interaction between RIG-I and 2B, while the 135- to 144-amino-acid region was not essential. We therefore speculated that 2B interacted with RIG-I through the 105- to 114-amino-acid region, and the 135–144-amino-acid region might have recruited and interacted with other proteins to form a complex to degrade RIG-I. The potential proteinases that can interact with 2B should be exploited to understand 2B-induced reduction of RIG-I.

In conclusion, the results of our experiments identified the antiviral role of RIG-I during FMDV infection and described for the first time a novel mechanism by which FMDV 2B evolves to induce a reduction of RIG-I and counteract the RIG-I-induced antiviral effect.

## References

[1] Alexandersen S, Mowat N 2005 Foot-and-mouth disease: host range and pathogenesis. Foot Mouth Dis Virus 288:9–42. doi:10.1007/3-540-27109-0_2. 15648173

[2] Grubman MJ, Baxt B 2004 Foot-and-mouth disease. Clin Microbiol Rev 17:465–493. doi:10.1128/CMR.17.2.465-493.2004. 15084510 PMC387408

[3] Nayak A, Goodfellow IG, Belsham GJ 2005 Factors required for the uridylylation of the foot-and-mouth disease virus 3B1, 3B2, and 3B3 peptides by the RNA-dependent RNA polymerase (3D^pol^) in vitro. J Virol 79:7698–7706. doi:10.1128/JVI.79.12.7698-7706.2005. 15919922 PMC1143669

[4] Moffat K, Howell G, Knox C, Belsham GJ, Monaghan P, Ryan MD, Wileman T 2005 Effects of foot-and-mouth disease virus nonstructural proteins on the structure and function of the early secretory pathway: 2BC but not 3A blocks endoplasmic reticulum-to-Golgi transport. J Virol 79:4382–4395. doi:10.1128/JVI.79.7.4382-4395.2005. 15767438 PMC1061540

[5] Moffat K, Knox C, Howell G, Clark SJ, Yang H, Belsham GJ, Ryan M, Wileman T 2007 Inhibition of the secretory pathway by foot-and-mouth disease virus 2BC protein is reproduced by coexpression of 2B with 2C, and the site of inhibition is determined by the subcellular location of 2C. J Virol 81:1129–1139. doi:10.1128/JVI.00393-06. 17121791 PMC1797538

[6] Moraes MP, Diaz-San Segundo F, Dias CC, Pena L, Grubman MJ 2011 Increased efficacy of an adenovirus-vectored foot-and-mouth disease capsid subunit vaccine expressing nonstructural protein 2B is associated with a specific T cell response. Vaccine 29:9431–9440. doi:10.1016/j.vaccine.2011.10.037. 22027486

[7] Jecht M, Probst C, Gauss-Muller V 1998 Membrane permeability induced by hepatitis A virus proteins 2B and 2BC and proteolytic processing of HAV 2BC. Virology 252:218–227. doi:10.1006/viro.1998.9451. 9875331

[8] Doedens JR, Kirkegaard K 1995 Inhibition of cellular protein secretion by poliovirus proteins 2B and 3A. EMBO J 14:894–907. 7889939 10.1002/j.1460-2075.1995.tb07071.x PMC398162

[9] van Kuppeveld FJ, Hoenderop JG, Smeets RL, Willems PH, Dijkman HB, Galama JM, Melchers WJ 1997 Coxsackievirus protein 2B modifies endoplasmic reticulum membrane and plasma membrane permeability and facilitates virus release. EMBO J 16:3519–3532. doi:10.1093/emboj/16.12.3519. 9218794 PMC1169977

[10] Matsumiya T, Stafforini DM 2010 Function and regulation of retinoic acid-inducible gene-I. Crit Rev Immunol 30:489–513. doi:10.1615/CritRevImmunol.v30.i6.10. 21175414 PMC3099591

[11] Kowalinski E, Lunardi T, McCarthy AA, Louber J, Brunel J, Grigorov B, Gerlier D, Cusack S 2011 Structural basis for the activation of innate immune pattern-recognition receptor RIG-I by viral RNA. Cell 147:423–435. doi:10.1016/j.cell.2011.09.039. 22000019

[12] Sadler AJ, Williams BR 2008 Interferon-inducible antiviral effectors. Nat Rev Immunol 8:559–568. doi:10.1038/nri2314. 18575461 PMC2522268

[13] Rice GI, Duany YD, Jenkinson EM, Forte GMA, Anderson BH, Ariaudo G, Bader-Meunier B, Baildam EM, Battini R, Beresford MW, Casarano M, Chouchane M, Cimaz R, Collins AE, Cordeiro NJV, Dale RC, Davidson JE, De Waele L, Desguerre I, Faivre L, Fazzi E, Isidor B, Lagae L, Latchman AR, Lebon P, Li CM, Livingston JH, Lourenco CM, Mancardi MM, Masurel-Paulet A, McInnes LB, Menezes MP, Mignot C, O'Sullivan J, Orcesi S, Picco PP, Riva E, Robinson RA, Rodriguez D, Salvatici E, Scott C, Szybowska M, Tolmie JL, Vanderver A, Vanhulle C, Vieira JP, Webb K, Whitney RN, Williams SG, Wolfe LA, Zuberi SM, Hur S, Grow YJ 2014 Gain-of-function mutations in IFIH1 cause a spectrum of human disease phenotypes associated with upregulated type I interferon signaling. Nat Genet 46:503–509. doi:10.1038/ng.2933. 24686847 PMC4004585

[14] Ahmad S, Hur S 2015 Helicases in antiviral immunity: dual properties as sensors and effectors. Trends Biochem Sci 40:576–585. doi:10.1016/j.tibs.2015.08.001. 26410598 PMC4584414

[15] Schmidt A, Endres S, Rothenfusser S 2011 Pattern recognition of viral nucleic acids by RIG-I-like helicases. J Mol Med 89:5–12. doi:10.1007/s00109-010-0672-8. 20820752

[16] Yoneyama M, Fujita T 2010 Recognition of viral nucleic acids in innate immunity. Rev Med Virol 20:4–22. doi:10.1002/rmv.633. 20041442

[17] Feng Q, Hato SV, Langereis MA, Zoll J, Virgen-Slane R, Peisley A, Hur S, Semler BL, van Rij RP, van Kuppeveld FJM 2012 MDA5 detects the double-stranded RNA replicative form in picornavirus-infected cells. Cell Reports 2:1187–1196. doi:10.1016/j.celrep.2012.10.005. 23142662 PMC7103987

[18] Deddouche S, Goubau D, Rehwinkel J, Chakravarty P, Begum S, Maillard PV, Borg A, Matthews N, Feng Q, van Kuppeveld FJM, Sousa CRE 2014 Identification of an LGP2-associated MDA5 agonist in picornavirus-infected cells. eLife 3:e01535. doi:10.7554/eLife.01535. 24550253 PMC3967861

[19] Barral PM, Sarkar D, Fisher PB, Racaniello VR 2009 RIG-I is cleaved during picornavirus infection. Virology 391:171–176. doi:10.1016/j.virol.2009.06.045. 19628239 PMC2743091

[20] Feng Q, Langereis MA, Olagnier D, Chiang C, van de Winkel R, van Essen P, Zoll J, Hiscott J, van Kuppeveld FJM 2014 Coxsackievirus cloverleaf RNA containing a 5′ triphosphate triggers an antiviral response via RIG-I activation. PLoS One 9:e95927. doi:10.1371/journal.pone.0095927. 24759703 PMC3997492

[21] Buskiewicz IA, Koenig A, Huber SA, Budd RC 2012 Caspase-8 and FLIP regulate RIG-I/MDA5-induced innate immune host responses to picornaviruses. Future Virol 7:1221–1236. doi:10.2217/fvl.12.115. 23503762 PMC3595017

[22] Kato H, Takeuchi O, Sato S, Yoneyama M, Yamamoto M, Matsui K, Uematsu S, Jung A, Kawai T, Ishii KJ, Yamaguchi O, Otsu K, Tsujimura T, Koh CS, Sousa CRE, Matsuura Y, Fujita T, Akira S 2006 Differential roles of MDA5 and RIG-I helicases in the recognition of RNA viruses. Nature 441:101–105. doi:10.1038/nature04734. 16625202

[23] Zheng H, Guo J, Jin Y, Yang F, He J, Lv L, Zhang K, Wu Q, Liu X, Cai X 2013 Engineering foot-and-mouth disease viruses with improved growth properties for vaccine development. PLoS One 8:e55228. doi:10.1371/journal.pone.0055228. 23372840 PMC3555929

[24] Lei CQ, Zhong B, Zhang Y, Zhang J, Wang SA, Shu HB 2010 Glycogen synthase kinase 3 beta regulates IRF3 transcription factor-mediated antiviral response via activation of the kinase TBK1. Immunity 33:878–889. doi:10.1016/j.immuni.2010.11.021. 21145761

[25] Zhou Q, Lin H, Wang S, Wang S, Ran Y, Liu Y, Ye W, Xiong X, Zhong B, Shu HB, Wang YY 2014 The ER-associated protein ZDHHC1 is a positive regulator of DNA virus-triggered, MITA/STING-dependent innate immune signaling. Cell Host Microbe 16:450–461. doi:10.1016/j.chom.2014.09.006. 25299331

[26] Cong L, Ran FA, Cox D, Lin S, Barretto R, Habib N, Hsu PD, Wu X, Jiang W, Marraffini LA 2013 Multiplex genome engineering using CRISPR/Cas systems. Science 339:819–823. doi:10.1126/science.1231143. 23287718 PMC3795411

[27] Li D, Yang W, Yang F, Liu H, Zhu Z, Lian K, Lei C, Li S, Liu X, Zheng H 2016 The VP3 structural protein of foot-and-mouth disease virus inhibits the IFN-β signaling pathway. FASEB J 30:1757–1766. doi:10.1096/fj.15-281410. 26813975

[28] Zhu ZX, Shi ZX, Yan WJ, Wei JC, Shao DH, Deng XF, Wang SH, Li BB, Tong GZ, Ma ZY 2013 Nonstructural protein 1 of influenza A virus interacts with human guanylate-binding protein 1 to antagonize antiviral activity. PLoS One 8:e55920. doi:10.1371/journal.pone.0055920. 23405236 PMC3566120

[29] Schmittgen TD, Livak KJ 2008 Analyzing real-time PCR data by the comparative C-T method. Nat Protoc 3:1101–1108. doi:10.1038/nprot.2008.73. 18546601

[30] Fuchsluger TA, Jurkunas U, Kazlauskas A, Dana R 2011 Corneal endothelial cells are protected from apoptosis by gene therapy. Hum Gene Ther 22:549–558. doi:10.1089/hum.2010.079. 21158568 PMC3081440

[31] Yang X, Ouyang HS, Chen FW, Pang DX, Dong MC, Yang SS, Liu XY, Peng ZY, Wang F, Zhang X, Ren LZ 2014 HMG-CoA reductase is negatively associated with PCV2 infection and PCV2-induced apoptotic cell death. J Gen Virol 95:1330–1337. doi:10.1099/vir.0.063644-0. 24643880

[32] Zhu ZX, Wei JC, Shi ZX, Yang YF, Shao DH, Li BB, Wang XD, Ma ZY 2013 Identification of human guanylate-binding protein 1 gene (hGBP1) as a direct transcriptional target gene of p53. Biochem Biophys Res Commun 436:204–211. doi:10.1016/j.bbrc.2013.05.074. 23727578

[33] Wang Y, Wang X, Li J, Zhou Y, Ho W 2013 RIG-I activation inhibits HIV replication in macrophages. J Leukoc Biol 94:337–341. doi:10.1189/jlb.0313158. 23744645 PMC3714567

[34] de Los Santos T, de Avila Botton S, Weiblen R, Grubman MJ 2006 The leader proteinase of foot-and-mouth disease virus inhibits the induction of beta interferon mRNA and blocks the host innate immune response. J Virol 80:1906–1914. doi:10.1128/JVI.80.4.1906-1914.2006. 16439546 PMC1367153

[35] Foeger N, Kuehnel E, Cencic R, Skern T 2005 The binding of foot-and-mouth disease virus leader proteinase to eIF4GI involves conserved ionic interactions. FEBS J 272:2602–2611. doi:10.1111/j.1742-4658.2005.04689.x. 15885108

[36] Kirchweger R, Ziegler E, Lamphear BJ, Waters D, Liebig HD, Sommergruber W, Sobrino F, Hohenadl C, Blaas D, Rhoads RE 1994 Foot-and-mouth disease virus leader proteinase: purification of the Lb form and determination of its cleavage site on eIF-4 gamma. J Virol 68:5677–5684. 8057448 10.1128/jvi.68.9.5677-5684.1994 PMC236969

[37] Hüsser L, Alves MP, Ruggli N, Summerfield A 2011 Identification of the role of RIG-I, MDA-5 and TLR3 in sensing RNA viruses in porcine epithelial cells using lentivirus-driven RNA interference. Virus Res 159:9–16. doi:10.1016/j.virusres.2011.04.005. 21539869

[38] Sato S, Li K, Kameyama T, Hayashi T, Ishida Y, Murakami S, Watanabe T, Iijima S, Sakurai Y, Watashi K, Tsutsumi S, Sato Y, Akita H, Wakita T, Rice CM, Harashima H, Kohara M, Tanaka Y, Takaoka A 2015 The RNA sensor RIG-I dually functions as an innate sensor and direct antiviral factor for hepatitis B virus. Immunity 42:123–132. doi:10.1016/j.immuni.2014.12.016. 25557055

[39] Weber M, Sediri H, Felgenhauer U, Binzen I, Banfer S, Jacob R, Brunotte L, Garcia-Sastre A, Schmid-Burgk JL, Schmidt T, Hornung V, Kochs G, Schwemmle M, Klenk HD, Weber F 2015 Influenza virus adaptation PB2-627K modulates nucleocapsid inhibition by the pathogen sensor RIG-I. Cell Host Microbe 17:309–319. doi:10.1016/j.chom.2015.01.005. 25704008 PMC4359673

[40] Loo YM, Fornek J, Crochet N, Bajwa G, Perwitasari O, Martinez-Sobrido L, Akira S, Gill MA, Garcia-Sastre A, Katze MG, Gale M 2008 Distinct RIG-I and MDA5 signaling by RNA viruses in innate immunity. J Virol 82:335–345. doi:10.1128/JVI.01080-07. 17942531 PMC2224404

[41] Ao D, Sun SQ, Guo HC 2014 Topology and biological function of enterovirus nonstructural protein 2B as a member of the viroporin family. Vet Res 45:87. doi:10.1186/s13567-014-0087-6. 25163654 PMC4155101

[42] de Jong AS, de Mattia F, Van Dommelen MM, Lanke K, Melchers WJG, Willems PHGM, van Kuppeveld FJM 2008 Functional analysis of picornavirus 2B proteins: effects on calcium homeostasis and intracellular protein trafficking. J Virol 82:3782–3790. doi:10.1128/JVI.02076-07. 18216106 PMC2268507

[43] Liu Y, Zhu Z, Zhang M, Zheng H 2015 Multifunctional roles of leader protein of foot-and-mouth disease viruses in suppressing host antiviral responses. Vet Res 46:127. doi:10.1186/s13567-015-0273-1. 26511922 PMC4625562

[44] Wang D, Fang L, Li K, Zhong H, Fan J, Ouyang C, Zhang H, Duan E, Luo R, Zhang Z, Liu X, Chen H, Xiao S 2012 Foot-and-mouth disease virus 3C protease cleaves NEMO to impair innate immune signaling. J Virol 86:9311–9322. doi:10.1128/JVI.00722-12. 22718831 PMC3416110

[45] Lawrence P, Schafer EA, Rieder E 2012 The nuclear protein Sam68 is cleaved by the FMDV 3C protease redistributing Sam68 to the cytoplasm during FMDV infection of host cells. Virology 425:40–52. doi:10.1016/j.virol.2011.12.019. 22280896

[46] Belsham GJ, McInerney GM, Ross-Smith N 2000 Foot-and-mouth disease virus 3C protease induces cleavage of translation initiation factors eIF4A and eIF4G within infected cells. J Virol 74:272–280. doi:10.1128/JVI.74.1.272-280.2000. 10590115 PMC111537

[47] Glaser W, Skern T 2000 Extremely efficient cleavage of eIF4G by picornaviral proteinases L and 2A in vitro. FEBS Lett 480:151–155. doi:10.1016/S0014-5793(00)01928-1. 11034318

[48] Ao D, Guo HC, Sun SQ, Sun DH, Fung TS, Wei YQ, Han SC, Yao XP, Cao SZ, Liu DX, Liu XT 2015 Viroporin activity of the foot-and-mouth disease virus nonstructural 2B protein. PLoS One 10:e0125828. doi:10.1371/journal.pone.0125828. 25946195 PMC4422707

[49] Han SC, Guo HC, Sun SQ, Jin Y, Wei YQ, Feng X, Yao XP, Cao SZ, Xiang Liu D, Liu XT 2016 Productive entry of foot-and-mouth disease virus via macropinocytosis independent of phosphatidylinositol 3-kinase. Sci Rep 6:19294. doi:10.1038/srep19294. 26757826 PMC4725844

[50] Wagner GG, McVicar JW 1970 Foot-and-mouth disease virus antibodies: comparison of a tissue culture microneutralization test with the assay in suckling mice. Appl Microbiol 20:995–997. 4321714 10.1128/am.20.6.995-997.1970 PMC377099

[51] Van kuppeveld FJ, Melchers WJ, Kirkegaard K, Doedens JR 1997 Structure-function analysis of coxsackie B3 virus protein 2B. Virology 227:111–118. doi:10.1006/viro.1996.8320. 9007064

[52] Harris JR, Racaniello VR 2005 Amino acid changes in proteins 2B and 3A mediate rhinovirus type 39 growth in mouse cells. J Virol 79:5363–5373. doi:10.1128/JVI.79.9.5363-5373.2005. 15827151 PMC1082767

